# Molecular Epidemiology of *Brucella abortus* in Northern Ireland—1991 to 2012

**DOI:** 10.1371/journal.pone.0136721

**Published:** 2015-09-01

**Authors:** Adrian Allen, Eleanor Breadon, Andrew Byrne, Thomas Mallon, Robin Skuce, Pauline Groussaud, Amanda Dainty, Judith Graham, Kerri Jones, Lorraine Pollock, Adrian Whatmore

**Affiliations:** 1 Agri-Food and Biosciences Institute (AFBI), AFBI Stormont, Belfast, United Kingdom; 2 School of Biological Sciences, Queens University, Belfast, United Kingdom; 3 OIE/WHO/FAO Reference Laboratory for Brucellosis, Department of Bacteriology, Animal and Plant Health Agency (APHA), New Haw, Addlestone, Surrey, United Kingdom; 4 Department of Agriculture and Rural Development, Veterinary Service, Belfast, Northern Ireland; Institut National de la Recherche Agronomique, FRANCE

## Abstract

**Background:**

Brucellosis is the most common bacterial zoonoses worldwide. Bovine brucellosis caused by *Brucella abortus* has far reaching animal health and economic impacts at both the local and national levels. Alongside traditional veterinary epidemiology, the use of molecular typing has recently been applied to inform on bacterial population structure and identify epidemiologically-linked cases of infection. Multi-locus variable number tandem repeat VNTR analysis (MLVA) was used to investigate the molecular epidemiology of a well-characterised *Brucella abortus* epidemic in Northern Ireland involving 387 herds between 1991 and 2012.

**Results:**

MLVA identified 98 unique *B*. *abortus* genotypes from disclosing isolates in the 387 herds involved in the epidemic. Clustering algorithms revealed the relatedness of many of these genotypes. Combined with epidemiological information on chronology of infection and geographic location, these genotype data helped to identify 7 clonal complexes which underpinned the outbreak over the defined period. Hyper-variability of some VNTR loci both within herds and individual animals led to detection of multiple genotypes associated with single outbreaks. However with dense sampling, these genotypes could still be associated with specific clonal complexes thereby permitting inference of epidemiological links. MLVA- based epidemiological monitoring data were congruent with an independent classical veterinary epidemiology study carried out in the same territory.

**Conclusions:**

MLVA is a useful tool in ongoing disease surveillance of *B*. *abortus* outbreaks, especially when combined with accurate epidemiological information on disease tracings, geographical clustering of cases and chronology of infection.

## Introduction

Bovine brucellosis, the most common bacterial zoonoses worldwide [1,2], caused by the bacterium *Brucella abortus*, is a major public and animal health issue worldwide. The disease can cause chronic and debilitating infection in humans and reproductive losses / abortion in cattle [3,4]. Aborted foetuses and amniotic fluids from infected animals are highly infectious [5] and are thought to be a major transmission route to new susceptible hosts [6]. The nasal and oral mucosae of animals are considered the most likely site of entry for the pathogen to new hosts [7,8].

The *B*. *abortus* bacterium is a facultative intracellular pathogen [9] but can remain viable in the environment for long periods provided there is an adequate moisture level. Conversely, strong sunlight and an arid climate can reduce the survival of the bacterium in the environment [7,9]. Further elucidation of the epidemiology of the pathogen has been gained by use of data from the greater Yellowstone area of the western United States where both elk and bison are known wildlife reservoirs. In this setting, animal density has been observed to play a crucial role in the likely outcome of a disease outbreak. Both elk and bison populations are at considerably greater risk of developing widespread infection if abortions occur during the winter period when grazing land is scarce leading to higher animal density [9]. In intensively farmed agricultural settings, such as Northern Ireland, dense populations of domesticated bovine animals are therefore at a considerable risk of becoming infected in the event of brucellosis mediated abortion within their herd.

Eradication of bovine brucellosis is undertaken using a test and slaughter protocol that makes use of internationally standardised bacterial culture and diagnostic tests. In addition, vaccination to agreed international standards is routinely deployed in some parts of the world [2, 10–12]. Whilst *B*. *abortus* eradication programmes have been effective in much of the developed world [13], including in Great Britain, the Republic of Ireland, Australia, New Zealand and most of North America [6,14], in many other locations, the disease remains a costly and challenging issue. Gaining and maintaining officially brucellosis free (OBF) status is economically desirable for nations since being free from disease increases herd productivity and opportunities for trade whilst reducing expenditure on control and/or eradication.

Despite initial success in disease eradication in Northern Ireland throughout the 1980s [15], three major sporadic outbreaks in 1997 [16] led to regional recrudescence [15]. The cost of the Northern Ireland eradication scheme between 1999 and 2013 has amounted to approximately £150 million sterling.

At the molecular level, the *Brucella* genus is characterised by a high level of nucleotide similarity [1,5,17]. The latter has, until relatively recently, been a handicap to effective characterisation of individual isolates for epidemiological tracing purposes [18]. However, recently developed molecular approaches permit the characterisation of individual tandem repeat sequences of DNA among *Brucella* isolates [5]. The genotyping of these rapidly evolving markers, by characterisation of the number of repeats at each locus, can be used to construct a genetic fingerprint / molecular type for bacterial isolates. These molecular types are well suited to investigating individual disease outbreaks and permit tracing of onward transmission in epidemiologically linked cases which share alleles at multiple tandem repeat loci [19]. Multiple variable number of tandem repeat (VNTR) loci can now be combined in multiplex assays which provide unprecedented levels of isolate discrimination within and between *Brucella* species, the latter is referred to as multi- locus VNTR analysis (MLVA) [5]. Bricker *et al*., [18] were the first to use such a technique with their highly discriminatory ‘HOOF-prints’ system. Since initial efforts using MLVA to distinguish isolates within *Brucella* species, alternative panels using different combinations of markers / loci have been proposed by various authors to improve discriminatory power and provide redundancy should some loci be monomorphic or hypervariable in certain sub-populations of the pathogen [4,20]. The latter may arise due to the mostly clonal nature of the *Brucella* species [5] and the related observation that there is considerable geographical structure among isolates in outbreaks [20]–such features can tend to lead to differing powers of discrimination for some loci in different populations. Thus, while use of a harmonised global MLVA scheme should be encouraged to facilitate understanding of international epidemiology [5] in some scenarios use of a scheme tailored to extant genetic diversity locally is required to maximally exploit the value of MLVA [5].

MLVA has recently been used to successfully monitor and inform on the epidemiology of *Brucella* infections in a variety of species such as cattle and wildlife in The United States of America [21], livestock and humans in Italy [22] and humans from across Europe [23,24] and China [25].

In the present study, during the recent Northern Ireland *B*. *abortus* epidemic, isolates from most confirmed infected animals were cultured and genotyped by MLVA. The panel of VNTRs examined was configured to maximise robust discrimination in the sampled population. In addition, MLVA was applied to multiple samples within animal, within-herd and between herd. The performance characteristics of this MLVA were evaluated and the molecular epidemiology of *B*. *abortus* monitored. This extensive and unique dataset, collected over a long time period, during an active epidemic, presented an excellent opportunity to assess the usefulness of MLVA when used in concert with conventional epidemiological techniques, to aid disease control measures.

## Materials and Methods

**Fig 1:**
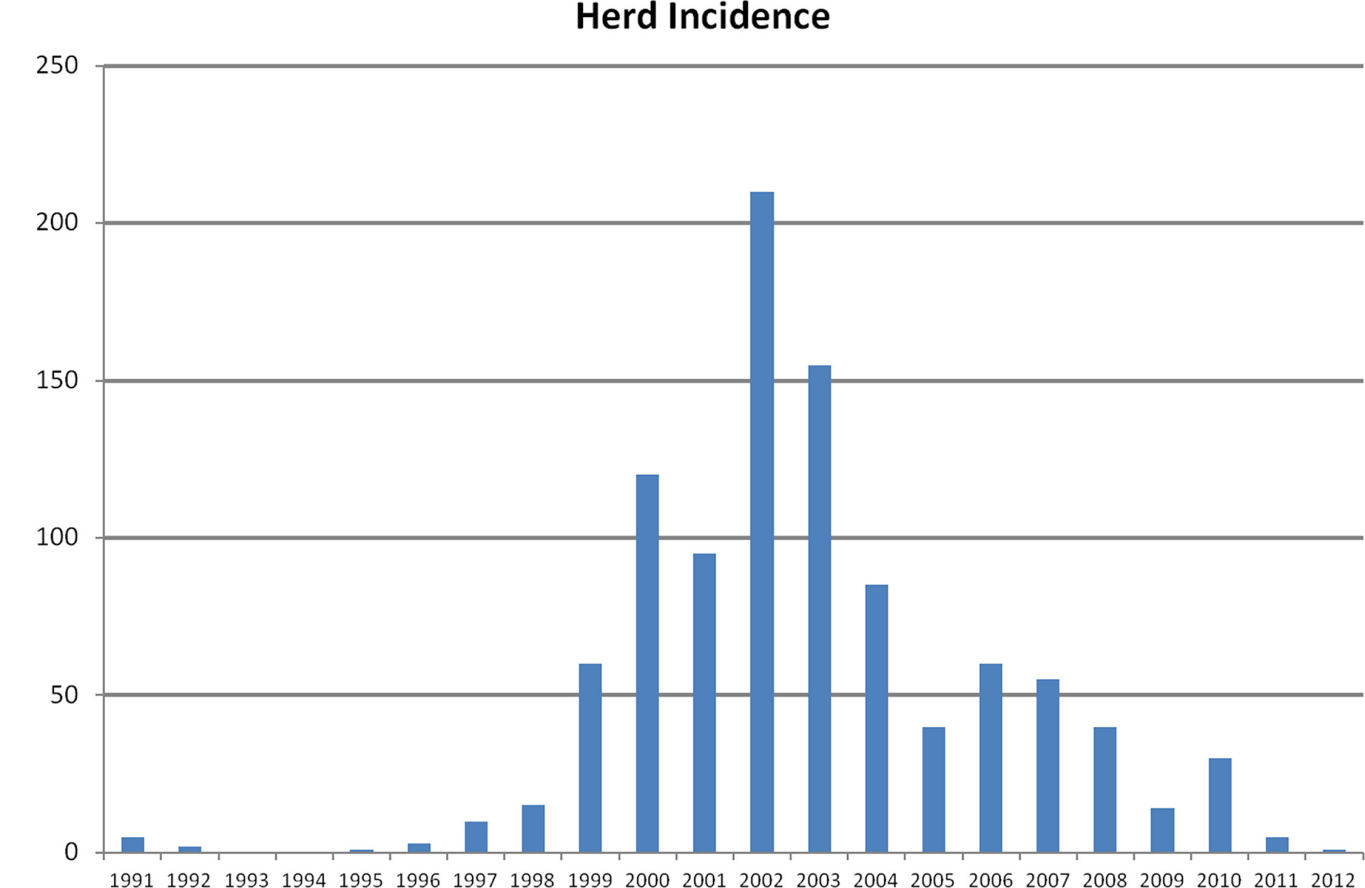
*Brucella abortus* confirmed herd incidence in Northern Ireland 1991–2012. http://www.dardni.gov.uk/index/statistics/animal-disease-statistics/statistics-brucellosis.htm

As the Northern Ireland brucellosis outbreak developed post 1997, herd and animal level incidences, as recorded by the Department of Agriculture and Rural Development (DARD) were observed to peak in January 2003 at 1.4% and 0.19%, respectively [26]. Since 2003/2004, there was a steady decrease in herd and animal incidence such that by August 2012, levels had reached 0.06% and 0.006%, respectively (Fig 1). To date, there have been no new laboratory confirmed cases of brucellosis since March 2012.

### *B*. *abortus* isolates sampling frame

**Fig 2:**
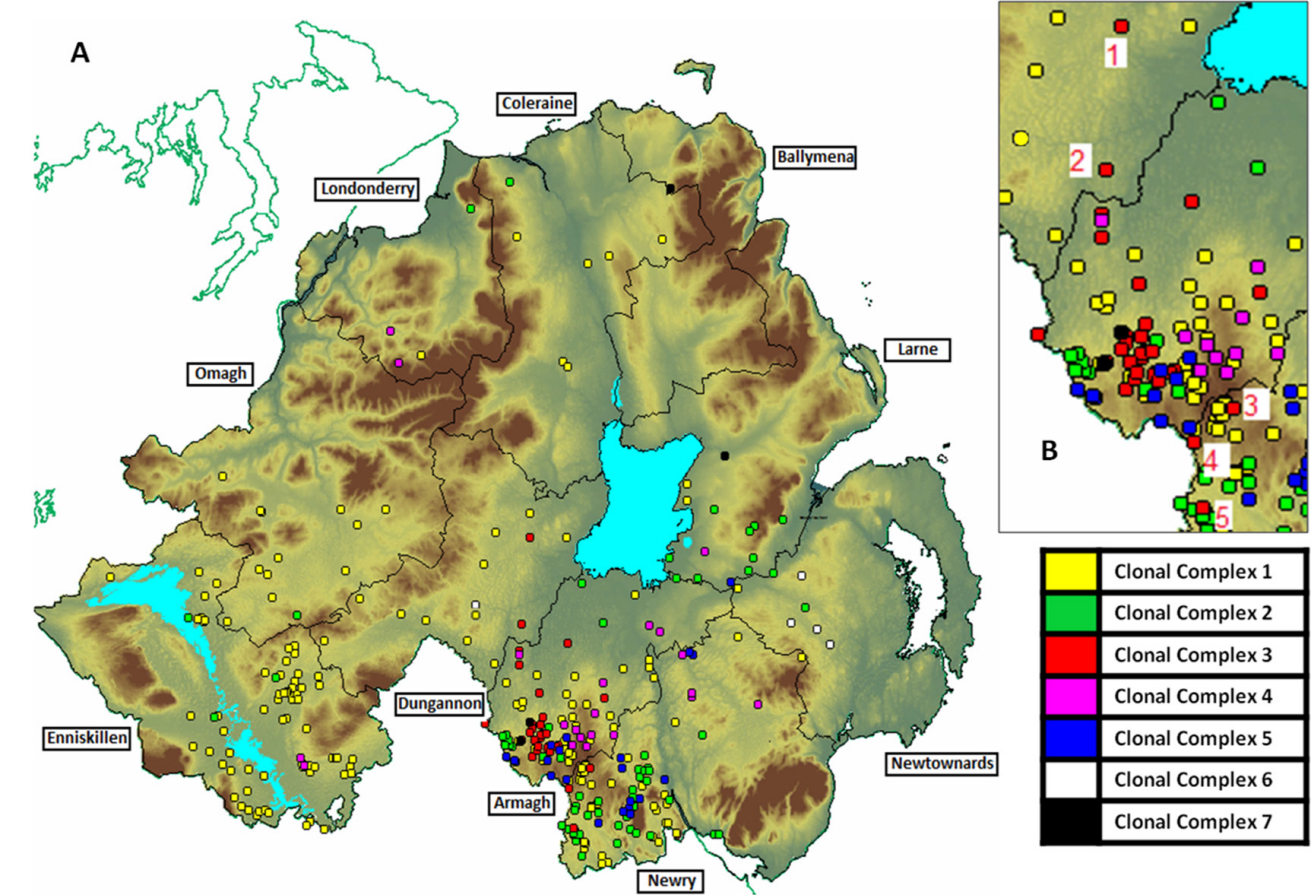
A—Geographic locations of first / disclosing isolates by Divisional Veterinary Office (DVO) and clonal complex designation. B–Inset of Armagh, Dungannon and Newry DVOs with 5 non DVO home range occurrences of Clonal Complex 3 highlighted.

During the period of the epidemic described above, all animals across all 10 Divisional Veterinary Offices in Northern Ireland (see Fig 2) were tested for brucellosis. The population and sub-populations of animals which tested positive for the disease and were analysed using molecular methods are detailed below.

#### Meta Population A

1270 isolates from 745 animals in 362 herds in Northern Ireland over the period of 1991 to 2012 were available to this study (see S1). Sub groups of this meta-population were used to address specific questions about the performance characteristics of MLVA.

#### Sub-population B

576 frozen isolates from 314 herds over the period Mar 1991–Aug 2008 were collected. These were MLVA typed by Animal Health and Veterinary Laboratories Agency (AHVLA) (MLVA26). In this group of samples 193 herds supplied 1 isolate and 121 herds supplied 2 or more isolates. Unfortunately, there is an under representation of samples in years 2001–2003 during the peak of the epidemic.

#### Sub-population C

Within sub-population B, the 386 disclosing isolates from each herd were used to determine the diversity indices for each of 26 VNTR loci tested for the purposes of determining which markers were optimal for discrimination of different molecular types–see details below.

#### Sub-population D

From Sept 2008—Mar 2012, 694 isolates from 182 cattle in 58 herds (including 58 disclosing isolates) were analysed at Agri Food and Biosciences Institute (AFBI) Stormont using an optimised panel of 11 VNTR markers. This multiple sampling of herds and individual animals provided an opportunity to assess within-herd and within-animal variability of the VNTR loci.

### *Brucella abortus* culture

*Brucella* infected animals were identified by serology and follow up laboratory confirmation by culture using standard methods as prescribed by the World Organisation for Animal Health [11]. Isolates were cultured from one or all of the following lymph node glands:- parotid, retropharyngeal, submandibular, supramammary/superficial inguinal, internal iliac. The glands were trimmed and macerated before being streaked onto a Farrell’s agar plate containing natamycin and incubated with 5–10% CO_2_ at 37°C for up to 14 days [27]. One colony was selected from the Farrell’s plate and sub-cultured onto another Farrell’s and a blood agar plate [11]. PCR-ready DNA was harvested by boiling a single colony from either the Farrell’s or blood agar plate in 200ul TE buffer at 100°C for 7 minutes, then centrifuging at 13, 000rpm for 10 mins. This boiled supernatant was used for all PCR (MLVA) assays.

### VNTR loci genotyping

AHVLA genotyped sub-populations B and C (576 sample set), including the disclosing isolates from 314 herds using the 21 VNTR loci described by Whatmore *et al*., [4] with an additional 5 VNTR loci (Bruce 19, 21, 27, 72 and 74) as described by Le Flèche *et al*., [20] resulting in a 26 VNTR MLVA test. A useful summary of MLVA loci and their repeat unit size, chromosome location and any aliases in nomenclature is provided by Scholz and Vergnaud [28]

Primer pairs consisted of the forward primer fluorescently labelled with either NED, 6-FAM or HEX and a reverse primer unlabelled. The 26 loci were multiplexed into 8 sets of three loci and 1 set of 2 loci. The PCR conditions were as previously described for multiplex PCR [4]. Amplified products were routinely diluted 1:50 in water and 1ul separated by capillary electrophoresis on an Applied Biosystems 3130xl genetic analyzer. All samples were run with Genescan Rox 500 (Applied Biosystems) size marker as an internal standard, and the bands were sized relative to these markers by using Genemapper software (Life Technologies). Raw base pair data for all repeats was recorded and assigned to discrete allele bins using Genemapper software. A standardised allele naming table was supplied by AHVLA based on the previous work of Bricker *et al* [18], Le Fleche *et al* [20] and Whatmore *et al* [4]. This table allowed for the binning of all PCR products, even those which exhibited 3’ partial repeats.

These initial data permitted the identification of a sub-set of 11 VNTRs, excluding those found to be monomorphic or extremely hypervariable, which were optimal for characterising the diversity of the *B*. *abortus* population sampled in Northern Ireland. Allele calls from each VNTR locus were concatenated, in order of increasing diversity index, to constitute an 11 MLVA molecular type. The remaining 694 isolates from 58 herds (sub-population D) were typed at AFBI using this MLVA11 assay.

The 11 loci were multiplexed into 3 sets of three loci and 1 set of 2 loci (See Table 1 for primer details). Each reaction mixture consisted of 1ul of 10 X Hot start PCR buffer (Qiagen), 2ul 5 xQ soln (Qiagen), 0.5ul of 10mM deoxynucleoside triphosphates, 0.4ul of 25mM MgCl2, 0.2ul of each of the 2 or 3 forward primers(10uM), 0.2ul of each of the 2 or 3 reverse primers (20uM) and 0.05ul of Hot Start *Taq* polymerase (Qiagen) in a final volume of 9.5ul of water followed by the addition 0.5ul of template DNA.

**Table 1:** MLVA11 primer sequences.

Locus	Fwd Primer Sequence	Reverse Primer Sequence
**Hoofprint 1**	GTGATTGCGGCGTGGTTCCGTTGAATGAG	TAGGGCARTARGGCAGTATGTTAAGGGAATAG
**Hoofprint 2**	CGCGCATGATCCGCGAACAGCTGGATG	GGGGAGTATGTTTTGGTTGCGCATGACCGC
**Hoofrpint 3**	CAGGCGCTTGAGGATGAGGCGGCAG	TAGGGCARTARGGCAGTATGTTAAGGGAATAG
**Hoofprint 4**	GCAGAATTTTCGAGGCATTCGGCGATG	TAGGGCARTARGGCAGTATGTTAAGGGAATAG
**Hoofprint 6**	GCCGCAGGAAAGCAGGCGATCTGGAGATTATC	TAGGGCARTARGGCAGTATGTTAAGGGAATAG
**Hoofprint 8**	GTGGGAAGCGTTATCCTTTAACGGGAGTAAGGG	GGGGAGTATGTTTTGGTTGCGCATGACCGC
**VNTR 2**	AGTTCGAGACCTTCATGGAA	CAGTTCCATTGCTTGCTGC
**VNTR 5a**	AATCACCCTTTTTCAGTCAAGG	GAAGAACATCTATTTCGCGGT
**VNTR 5b**	GTGGTGGACAAGGCAAGTA	CGCGAGGTTTTCGGCCAGA
**VNTR 16**	GAATGATAAGCTTCACCTGAATA	CGCGTTTCGATTGTGGAAA
**BRUCE 72** *	GAAGACGGCTATCGACTGGTCT	GTTTCAATGAAGGCGAGGTGAG

* indicates a locus not included in the originally described VNTR21 scheme [4]but taken from the panel described by Le Fleche *et al* 2006 characterising 80 repetitive loci [20].

The PCR cycling parameters were 95°C for 15 min; followed by 35 cycles of 95°C for 30sec., 58°C for 30sec., and 72°C for 1 min; then a final extension time of 20 min. Post PCR 1ul of amplified product was run on an Applied Biosystems 3130xl genetic analyzer as described above, and analyzed using Genemapper software (Life Technologies). Four positive controls were run in PCR reactions–AHVLA *B*. *abortus* 544 biovar 1, AHVLA *B*. *melitensis* 16M biovar 1, AHVLA *B*. *suis* 1330 biovar 1 and AFBI *B*. *abortus* 86/8/59 biovar 2. A panel of 7% repeats was performed by AFBI to compare with results generated by AHVLA to ensure consistent technology transfer.

### Epidemiological Data

Data on chronology of detection of infection and geographical location of herd breakdown were made available from the Animal and Public Health Information database (APHIS) [29]. These data were compiled for all isolates for the purposes of monitoring the epidemic, but additionally, to provide insight into those outbreaks which were potentially related when combined with molecular typing data.

### Genotypic Data Analysis

The genetic diversity of each VNTR locus in all sub groups of the 1270 total isolates collected was determined using the Hunter-Gaston diversity index (DI) [30]. The DI was calculated using the V-DICE program from the HPA-Bioinformatics tools at http://www.hpa.org.uk. The DI can range from zero (monomorphic) to one and is the probability of randomly drawing two samples from a population which exhibit a different allele at the locus in question.

Cluster analysis was undertaken to determine the potential relatedness of the concatenated 11 VNTR *Brucella* genotypes. A dendrogram of all 98 disclosing isolate genotypes was produced using VNTR allele calls imported into the Bionumerics software package, (v 6.6, Applied Maths, Belgium), and analyzed as character types. Cluster analysis was performed using the categorical coefficient, implying character sets are unordered, and the unweighted pair group method using arithmetic averages (UPGMA). All markers were given equal weightings. Dendrogram construction was undertaken using only MLVA data with no additional epidemiological data on location or chronology of infection utilised. In addition, the standard minimum spanning tree (MST) approach offered by eBurst [31] was applied to the total 387 herd level disclosing isolate samples collected over the whole sampling period. Criteria for membership of a cluster were set to isolates exhibiting identical genotypes at 9 of the 11 genotyped loci, with single locus and double-locus variants of nearest neighbours accepted as members of clonal complexes. The default 1000 bootstraps were undertaken to identify the potential founder molecular type of each of the clonal complexes. Combined genotype and epidemiological data were used to define the relatedness of isolate genotypes and to assign membership of clonal complexes. Specifically, those isolates which exhibited an identical or closely related genotype (permitting single and double locus variants), occurring contemporaneously in close geographical proximity were classified as belonging to the same clonal complex. Isolates falling outside of home range clusters were assigned to the same clonal complex as that observed in the cluster, provided close genotypic similarity to isolates within the cluster was observed and the contemporaneous nature of infection was established. In addition, on some occasions, sporadic non clustered isolates could also be assigned a clonal complex on the basis of exhibiting an identical or closely related genotype to a brucellosis affected herd from which the farm in question had recently purchased animals. Herds were deemed to be epidemiologically linked whenever they were observed to possess isolates from the same clonal complex.

### Spatio temporal analyses

First isolates from sub-population C, across the duration of the epidemic, had their herd co-ordinates entered into ArcGIS for geographical clustering analysis. Clustering was assessed using nearest neighbour analysis and the Multi-Distance Spatial Cluster Analysis tool, based on Ripley's K-function [32], with a confidence envelope constructed around expected values using 99 simulations.

Herd breakdowns were defined on the basis of the following criteria:

1—If after 12 months there had been no additional confirmed reactor animals identified, a herd was presumed to be clear of infection. This 1 year period provided opportunity for all pregnant cattle to produce calves. If no new abortion events had occurred in this period, or any culture confirmed *Brucella* reactors had been identified, then it was assumed that infection had been cleared.

2—Using pathogen molecular data could further refine criterion 1. Sharing the same clonal complex (see below) of strain separated by at least a year of no intermittent confirmed reactors or abortions was not considered reason enough to suggest both disease occurrences were in reality the same event. Since multiple genotypes exist within clonal complexes, it was difficult to say that two events were definitively linked over the elapsed time period. If however clonal complex and isolate genotype were the same across any period of time within the same herd, this was taken as evidence of an ongoing infection event.

Single isolates per animal from sub-population B presented an opportunity to, within each confirmed herd brucellosis breakdown, answer two specific questions:

1—What factors affected metrics of strain diversity during herd episodes?

Number of strains per episode was highly skewed, and was therefore simplified to a binary outcome (0 = diversity index of 1; 1 = diversity index>1) for modelling purposes. This diversity outcome variable was modelled using a maximum likelihood logistic regression model. As there was clustering within the dataset (animals–clustered within herds), we adjusted our model using the clustering sandwich estimator within Stata 11 (College Station, TX: StataCorp). This estimator relaxes the requirement of independence within groups (intragroup correlation), but treats groups independently (no intergroup correlation dependence). This clustering adjustment affects the variance-covariance matrix and standard errors but not the parameter estimates. Diversity was modelled against independent variables: clonal complex (categorical; 8 levels), time to clear infection (modelled as a binary dummy variable, 0 meaning 0 days; 1 meaning >0 days to clear) and number of positives disclosed during the episode.

2—What factors influence the time to clearance?

Time to clearance was also highly skewed in the dataset and was consequently also modelled as a binary outcome variable of time (0 = whether the herd cleared immediately, 1 = Clearance time). This outcome variable was modelled against independent variables clonal complex and the number of positives within the herd during an episode using a logistic regression model.

The multiple isolates per animal from Sub Population D were similarly used to assess the impact on pathogen diversity, within herd breakdowns, of number of infected sites sampled per animal. Correlation analysis was used in STATA 11 to address this question and by using a Poisson model of the count of strains, related to the number of sites sampled per animals, while controlling for clustering within herd.

## Results

### Discriminatory Power of VNTR loci in the Northern Irish *B*. *abortus* Population

Diversity indices for all individual markers used across meta population A and sub-populations B, C and D are shown in Table 2.

**Table 2:** Individual and 11 VNTR combined loci discrimination indices (DI) 95% confidence intervals and number of loci observed in study meta population and sub-populations.

Locus Identity	Meta Population A (1270 isolates)DI	Meta Population ANo. of loci	Sub population B (576 isolates)DI	Sub population B No. of loci	Sub population C (314 disclosing isolates)DI	Sub population C No. of loci	Sub population D (694 isolates)DI	Sub population D No. of loci
**Hoofprint 8**	0 (0.000–0.006)	1	0(0.000–0.013)	1	0(0.000–0.022)	1	0(0.000–0.011)	1
**VNTR 16**	0.002 (0.000–0.005)	2	0.003(0.000–0.010)	2	0.006(0.000–0.018)	2	0(0.000–0.011)	1
**Hoofprint 6**	0.009 (0.002–0.017)	2	0.021(0.004–0.037)	2	0.03(0.004–0.056)	2	0(0.000–0.011)	1
**Hoofprint 2**	0.031(0.018–0.045)	5	0.058(0.031–0.084)	5	0.048(0.016–0.080)	4	0.009(0.000–0.018)	4
**VNTR 5a**	0.107(0.085–0.130)	4	0.22(0.179–0.261)	4	0.191(0.138–0.243)	2	0(0.000–0.011)	1
**VNTR 2**	0.242(0.214–0.270)	4	0.437(0.405–0.469)	4	0.451(0.408–0.493)	4	0(0.000–0.011)	1
**BRUCE 72** *	0.232(0.201–0.263)	10	0.458(0.408–0.507)	10	0.496(0.432–0.560)	10	0.006(0.000–0.014)	2
**Hoofprint 1**	0.233(0.202–0.264)	11	0.461(0.411–0.510)	11	0.497(0.433–0.562)	11	0.006(0.000–0.014)	2
**VNTR 5b**	0.373(0.342–0.404)	10	0.588(0.557–0.619)	10	0.616(0.576–0.655)	10	0.089(0.060–0.118)	4
**Hoofprint 4**	0.519(0.491–0.547)	7	0.678(0.656–0.699)	6	0.673(0.639–0.706)	6	0.131(0.097–0.165)	6
**Hoofprint 3**	0.742(0.724–0.759)	11	0.739(0.716–0.763)	11	0.715(0.679–0.751)	11	0.542(0.504–0.579)	7
**VNTR 12b**	N/A	N/A	0.813(0.798–0.827)	15	0.822(0.807–0.838)	13	N/A	N/A
**VNTR 12a**	N/A	N/A	0.840(0.825–0.855)	18	0.844(0.824–0.863)	15	N/A	N/A
**Hoofprint 7**	N/A	N/A	0.875(0.866–0.885)	22	0.867(0.852–0.882)	14	N/A	N/A
**BRUCE 21** *	N/A	N/A	0.007(0.000–0.016)	3	0.006(0.000–0.018)	2	N/A	N/A
**Hoofprint 5**	N/A	N/A	0(0.000–0.013)	1	0(0.000–0.022)	1	N/A	N/A
**VNTR 7**	N/A	N/A	0(0.000–0.013)	1	0(0.000–0.022)	1	N/A	N/A
**VNTR 14**	N/A	N/A	0(0.000–0.013)	1	0(0.000–0.022)	1	N/A	N/A
**VNTR 17**	N/A	N/A	0(0.000–0.013)	1	0(0.000–0.022)	1	N/A	N/A
**VNTR 21**	N/A	N/A	0(0.000–0.013)	1	0(0.000–0.022)	1	N/A	N/A
**VNTR 24**	N/A	N/A	0(0.000–0.013)	1	0(0.000–0.022)	1	N/A	N/A
**VNTR 26**	N/A	N/A	0(0.000–0.013)	1	0(0.000–0.022)	1	N/A	N/A
**VNTR 27**	N/A	N/A	0(0.000–0.013)	1	0(0.000–0.022)	1	N/A	N/A
**BRUCE 19** *	N/A	N/A	0(0.000–0.013)	1	0(0.000–0.022)	1	N/A	N/A
**BRUCE 27** *	N/A	N/A	0(0.000–0.013)	1	0(0.000–0.022)	1	N/A	N/A
**BRUCE 74** *	N/A	N/A	0(0.000–0.013)	1	0(0.000–0.022)	1	N/A	N/A
**11 VNTR combined**	0.839 (0.820–0.858)	N/A	0.954(0.947–0.962)	N/A	0.964(0.957–0.971)	N/A	0.634(0.597–0.670)	N/A

* indicates a locus not included in the originally described VNTR21 scheme [4]but taken from the panel described by Le Fleche *et al* 2006 characterising 80 repetitive loci [20].

A combined MLVA11 diversity index is also shown in Table 2 for each of the population samples described above.

From molecular typing data for the initial 314 disclosing isolates (sampled 1991–2008), several markers were sub-optimal for use in the Northern Irish population because they were mono-morphic. These 12 loci were Hoofprint 5, VNTR 7, VNTR 14, VNTR 17, VNTR 21, VNTR 24, VNTR 26, VNTR 27, BRUCE 19, BRUCE 21, BRUCE 27 and BRUCE 74. These loci were subsequently not used for further analyses (See Table 2 columns 6 and 7).

Hoofprint 8 was also monomorphic for the ‘2’ allele. Previous work has shown that this locus mutates so slowly that the 2 allele has become fixed in *B*. *abortus* specific species of *Brucella* bacteria [18]. Our data appear to support this conclusion. Consequently, Hoofprint 8 was retained in our assay as a proxy measure of “species” identification.

Conversely, VNTR12a, VNTR12b and Hoofprint 7 were excluded from the final genotyping panel despite exhibiting high DI values. All three loci appeared to exhibit hypervariability. For these loci, the diversity indices and / or number of loci exhibited in the 576 isolates of sub-population B was greater than in the 314 disclosing isolates from sub-population C (see columns 4–7 of Table 2). Since sub-population B contains multiple samples from the same herd, this finding suggests that within herds, isolates from multiple animals displayed considerable allelic variability at these loci. Such instability was considered to limit the usefulness of these loci in epidemiological tracings.

The final panel of 11 loci used were Hoofprint 1, 2, 3, 4, 6 and 8, VNTRs 2, 5a, 5b and 16, and Bruce 72.

### MLVA11 of herd level isolate genotypes

From the 314 disclosing isolates collected over the entire sampling period (1991–2012), a total of 98 unique MLVA profiles were observed. These profiles, their clonal complex assignation and frequency over the entire period of the outbreak (1991–2012) are detailed within S1 Dataset.

**Fig 3:**
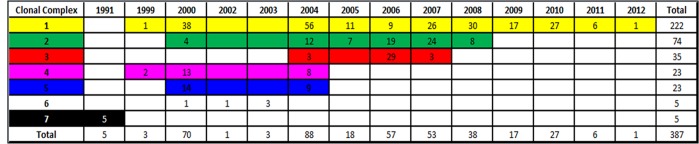
Frequencies of all disclosing isolates by assigned strain clonal complex and year of outbreak.

Geographic locations of each associated breakdown are shown in Fig 2. Frequencies of each 1^st^ isolate clonal complex over the individual years making up the period of the study are detailed in Fig 3.

Frequencies of isolates from each clonal complex by year of outbreak and location (DARD Divisional Veterinary Office DVO) are shown in Table 3.

**Table 3:** Clonal complex disclosing isolate frequencies by year of outbreak and geographical location (DARD DVO). Percentage breakdown of each Clonal Complex by DVO also included.

	1991	1999	2000	2002	2003	2004	2005	2006	2007	2008	2009	2010	2011	2012	Total	% of Total
Clonal Complex 1		1	38			56	11	9	26	30	17	27	6	1	222	100.0
Armagh		1	14			3			4	7	2	13	2	1	47	21.2
Coleraine							1		3	2	2				8	3.6
Derry										1					1	0.5
Dungannon			2			4			3	1	2	1			13	5.9
Enniskillen			14			32	10	8	12	4					80	36.0
Larne			1						1	1					3	1.4
Newry			6			5			2	14	10	13	4		54	24.3
Newtownards			1			1									2	0.9
Omagh						11		1	1		1				14	6.3
Clonal Complex 2			4			12	7	19	24	8					74	100.0
Armagh						2		7	3	3					15	20.3
Coleraine			1												1	1.4
Derry			1												1	1.4
Enniskillen			1			1	1		2						5	6.8
Larne			1			5	1		3						10	13.5
Newry						2	5	12	16	4					39	52.7
Newtownards						2									2	2.7
Omagh										1					1	1.4
Clonal Complex 3						3		29	3						35	100.0
Armagh						3		25	2						30	85.7
Dungannon								2							2	5.7
Newry								2	1						3	8.6
Clonal Complex 4		2	13			8									23	100.0
Armagh		2	8			4									14	60.9
Derry			2												2	8.7
Enniskillen			2												2	8.7
Larne			1												1	4.3
Newry						4									4	17.4
Clonal Complex 5			14			9									23	100.0
Armagh			6			3									9	39.1
Larne						1									1	4.3
Newry			8			5									13	56.5
Clonal Complex 6			1	1	3										5	100.0
Dungannon			1												1	20.0
Newtownards				1	3										4	80.0
Clonal Complex 7	5														5	100.0
Armagh	3														3	60.0
Ballymena	1														1	20.0
Larne	1														1	20.0
**Grand Total**	**5**	**3**	**70**	**1**	**3**	**88**	**18**	**57**	**53**	**38**	**17**	**27**	**6**	**1**	**387**	

Of note is the finding that 3 of the 98 MLVA profiles observed, genotypes 110, 66 and 37 were also characterised as biovar 2 by classical biotyping. All other isolates were biovar 1.

### MLVA11 within herd isolates

From the 694 isolates of sub-population D collected from 2008 to 2012, 30 (52%) of the 58 herds represented exhibited detection of *B*. *abortus* infection in two or more animals. In 20 (67%) of these 30 herds, more than one molecular type was associated with the outbreak. The locus which varied most was Hoofprint 3, accounting for all variable genotypes.

### MLVA11 within animal isolates

The 694 isolates of sub-population D were taken from 182 individual animals. Up to five lymph node extracts from these animals could be used for the confirmation of *B*. *abortus*. In 32 animals, only one site was culture positive. In the remaining 150 animals, between 2 and 5 sites were culture positive. Of this 150 multiple isolate subset of animals, 43 (29%) exhibited more than 1 molecular type, varying at one or more loci. The loci which varied most were Hoofprint 3 and Hoofprint 4, accounting for 34 (79%) of the 43 variable genotypes.

In addition, 23 animals were sampled on two occasions, once by vaginal swab (VS), and secondly post mortem at a secondary site such as lymph nodes. In 9 (39%) of these two sample pairs, differences in allelic state were observed between first and second isolates at either Hoofprint 3, Hoofprint 4, or both loci.

### Geographic Clustering of isolates

Herd locations were plotted using MAPINFO 7.5 (Pitney Bowes) as illustrated in Fig 2. Molecular types of high allelic similarity (single locus and dilocus variants) which occurred contemporaneously and clustered in specific geographic locations were considered to be epidemiologically linked and therefore designated as belonging to a specific clonal complex. Nearest neighbour analyses indicated there was significant clustering over the duration of the epidemic for disclosing isolates from Clonal Complexes 1, 2, 4 and 5 (Z-scores <-3; P<0.001), with shorter average nearest neighbour distances in comparison to a homogenous (random) distribution. Clonal complex 3 had a distribution not significantly different to a random distribution (Z-score = -0.277; p = 0.782). In contrast, complexes 6 and 7 were considered more dispersed than a random distribution (Z-scores>3; P<0.001), however caution should be applied to these latter results as there were only 5 observations for each complex respectively. From the Ripley’s K function analysis, the observed K-distance statistic of clonal complexes 1–5 were greater than the expected K-statistic for each of the ten distance bands estimated. In all cases, for clonal complexes 1–5, the observed K statistic was greater than the upper estimate of the confidence envelope, indicating that these patterns were significantly clustered. In clonal complexes 6 and 7, there was evidence of both clustering (observed >K than expected) and dispersion (observed<K than expected) depending on the spatial scale. However, these deviations from the expected K from a random point process were never significant as the observed K were within the confidence envelope estimates. Again, one should note that the numbers of disclosing isolates available for analysis, in from clonal complexes 4–7, and in particular 6 and 7 are below 30 which may lead to spurious results. Outputs from nearest neighbour analyses and Ripley’s K function are shown in S2 Dataset and S1 Table.

### Cluster Analysis by Dendrogram and e-Burst

**Fig 4:**
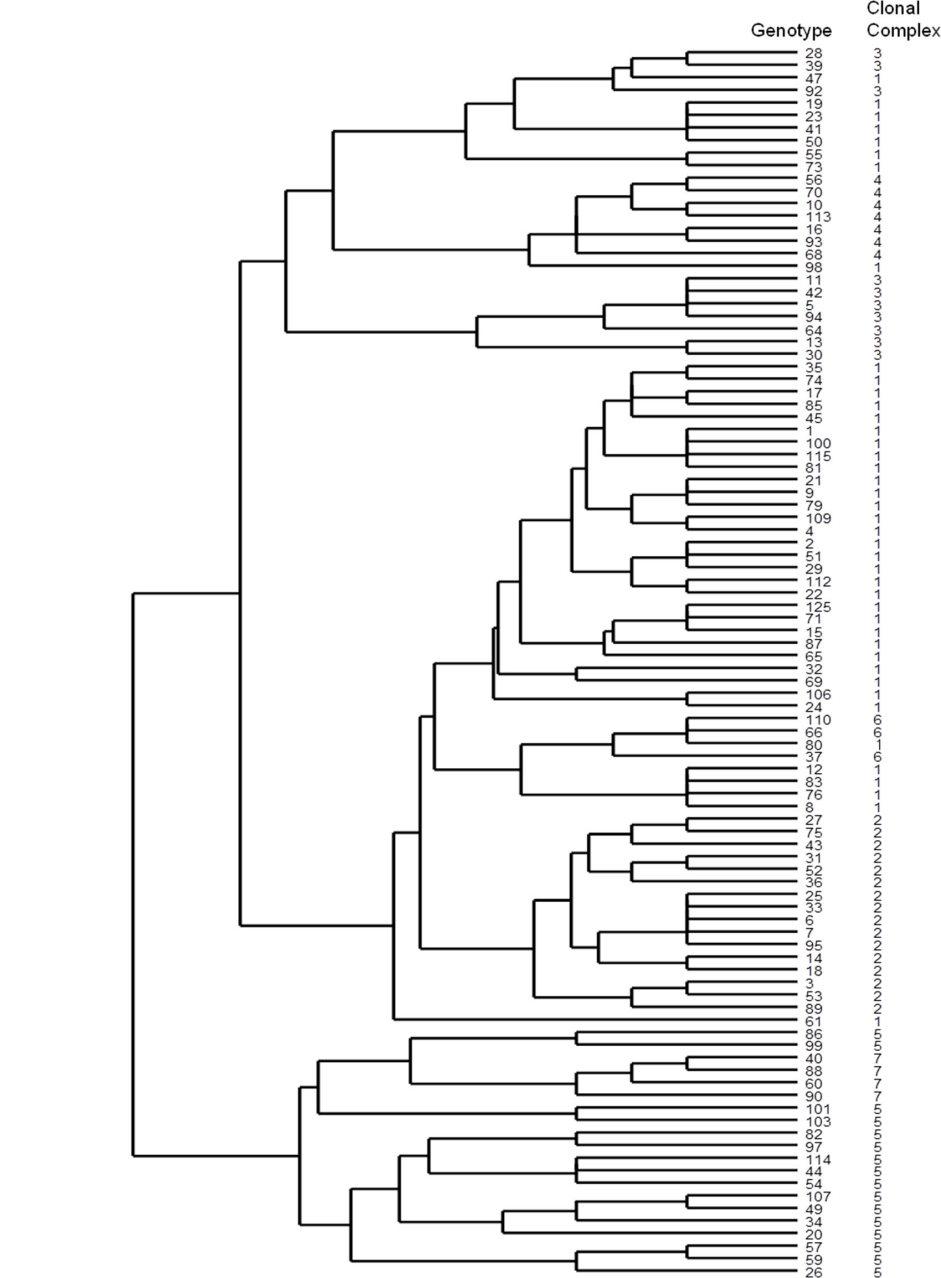
Dendrogram of 98 1^st^ isolate genotypes. Strain genotype and clonal complex assignation indicated.

The dendrogram of genetic relatedness of all 98 disclosing isolate genotypes is shown in Fig 4. Observed clustering was produced using only MLVA data–no additional epidemiological data were used. *B*. *abortus* genotypes appear to separate into two major lineages over the 1991–2012 period, with multiple sublineages observed in each.

**Fig 5:**
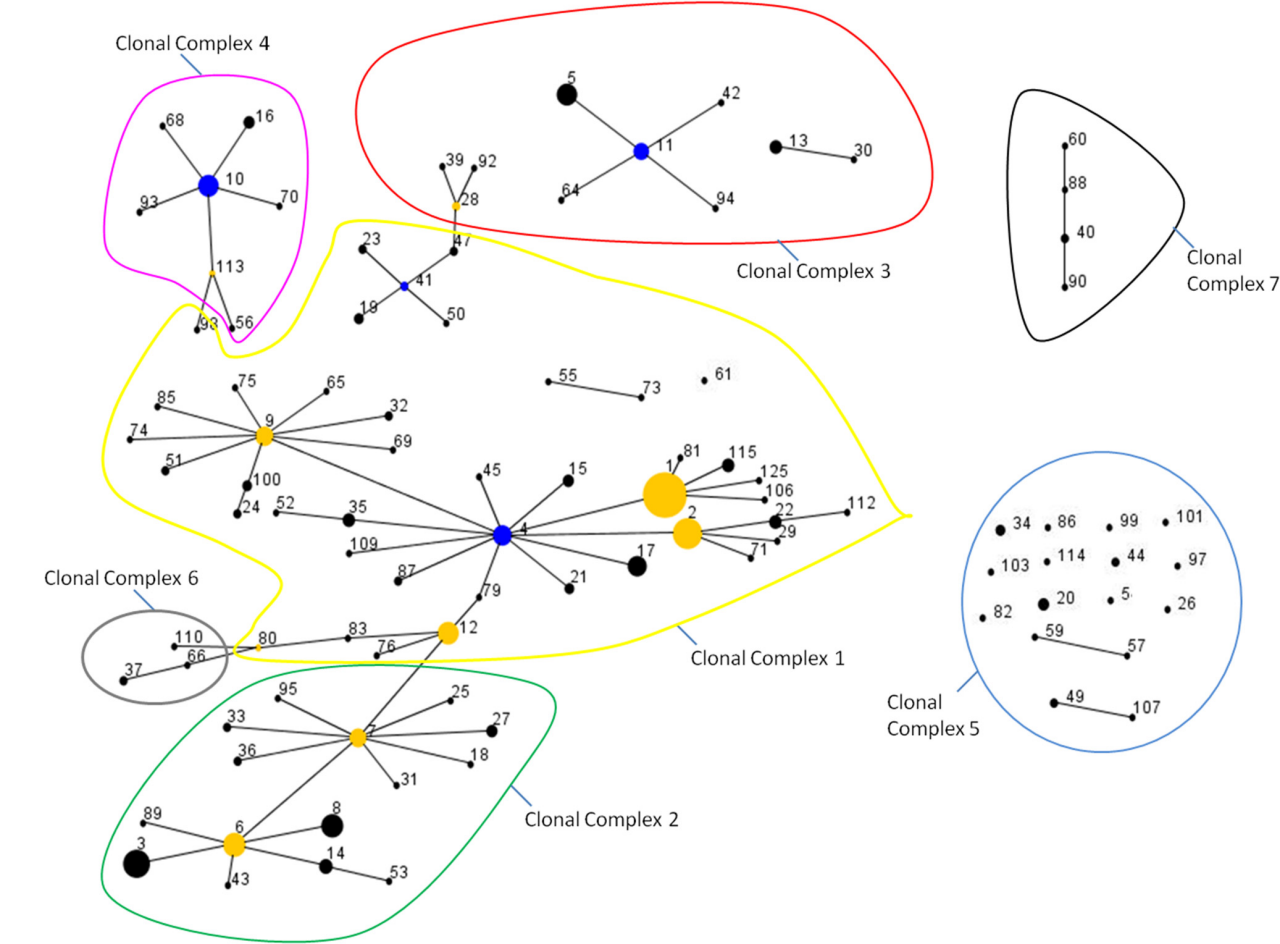
eBurst Minimum Spanning Trees of 98 disclosing isolate MLVA11 genotypes. Clonal Complex designation based on genotype similarity and epidemiological information indicated. Blue nodes indicate putative founder genotypes for a complete cluster. Yellow nodes indicate putative founders of sub clusters within larger clusters. Node size is related to frequency of observance of a specific genotype. Lines connecting node pairs are indicative of single locus variation.

Relatedness of disclosing isolate genotypes as assessed by MST analysis in e-Burst is shown in Fig 5. Isolates which exhibited a high degree of allelic similarity at all loci clustered together in closely related clonal complexes when combined with epidemiological data on location and chronology of infection. These clusters consist of a major genotype accounting for multiple isolates (indicated by size of the circle defining the genotype), surrounded by closely related single and double-locus variants which accounted for fewer isolates.

One large cluster of closely related genotypes (see Fig 5) accounted for 52 of 98 genotypes (53%) and 287 of 387 isolates (74%) observed among the disclosing sample sub-group. An additional 8 clusters accounted for 33 (34%) genotypes and 81 (21%) isolates. 13 genotypes, represented by just one isolate each (13% of genotypes, 3% of isolates), were not clustered by eBurst.

### Clonal complex assignments

By using combined epidemiological information and clustered genotype data, we determined that over the study period, seven *B*. *abortus* clonal complexes existed. These clonal complex designations are shown within S1 Dataset and colour coded on Figs 2, 3 and 5.

#### Pathogen diversity analyses

Analysis of the single animal isolates from Sub Population B 1991–2008 –across all 314 herds from this sub-population, 328 herd breakdown events were identified. Of these, 300 were single infection events. 28 events were observed to be the result of two breakdown occurrences in 14 of the herds. 260 of the herd breakdown events were cleared in one eradication event, with 68 taking a longer period to purge detectable infection from the farm. In these cases, time to clear varied from 1 day to 685 days from initial disclosure (See S1 Dataset). Time to clear was highly skewed with a mean time of 56 days and a standard deviation of 125. In addition, 312 of breakdown events involved only a single animal, with the remaining 68 breakdown events involving between 2 and 13 animals—mean 1.7 animals, standard deviation 1.4 (See S1 Dataset). 283 of the breakdowns involved just a single strain genotype, whilst 45 involved between 2 and 4 genotypes–mean 1.2 genotypes, standard deviation 0.5 (see S1 Dataset).

Statistical analysis indicated that within breakdown events, Clonal Complex did not affect strain diversity (Wald test χ^2^ (df: 3) = 3.61; Prob > χ^2^ = 0.307). Consequently, Clonal Complex was dropped from the analysis and the model refitted without it. Refitting revealed that the number of positives within a herd was significantly and positively related to the probability of a herd having a diverse strain infection during an episode. With each unit increase (each animal positive) the odds of a infection being diverse increased by 3.4 (SE: 1.03; P<0.001). The time to clear infection was not significant in this model (p = 0.242). However, in a uni-variable analysis, time was significant (OR: 11.3; SE: 4.0; p<0.001). Episodes that failed to clear infection immediately had an odds ratio of 11.3 of being a diverse infection episode. There was a strong correlation between time and the number of positives (r = 0.58), indicating that these variables were not independent of each other.

Statistical analysis also indicated that Clonal Complex did not affect time to clear infection from a herd (overall Wald test: χ^2^ (df: 5) = 5.47; Prob > χ^2^ = 0.361). This predictor was then dropped, and the model refitted. The refitted model indicated that the length of time it took to clear an infection was significantly affected by the number of positive animals that were detected (OR: 4.71; p<0.001).

Analysis of the animal isolates from sub-population D–Of the 182 individual animals represented in sub-population D, 150 had been sampled from more than one infected site. The mean number of sites sampled across the whole sub-population was 3.8 (standard deviation 1.6). 139 of the 182 animals samples exhibited only 1 genotype, with the remaining 43 animals exhibiting between 2 and 4 strains. Mean number of strains isolated per animal was 1.3 (standard deviation 0.57). Analysis identified a weak but significant correlation (r = 0.31; P< 0.001) between the number of sites sampled and genotype diversity. A Poisson model suggested that for every unit increase in number of sites tested, there was a corresponding increase of 9% in pathogen diversity within animals (exp(β) = 1.094).

## Discussion

### Discriminatory Power of MLVA loci in Northern Irish *B*. *abortus* Population

After removal of monomorphic and hypervariable VNTR loci detected in the survey populations described above, the following eleven VNTR loci were optimal for robust identification and characterisation of *B*. *abortus* in Northern Ireland: Hoofprint 8, VNTR 16, Hoofprint 6, Hoofprint 2, VNTR 5a, VNTR 2, BRUCE 72, Hoofprint 1, VNTR 5b, Hoofprint 4 and Hoofprint 3. Genotypes were recorded as concatenated strings of tandem repeat numbers with the loci arranged in order of increasing diversity (left to right).

### Clustering and Designation of strain clonal complexes

The minimum spanning trees produced by eBurst conservatively clustered closely related MLVA11 profiles (see Fig 5). When combined with epidemiological data, geographical clustering and period of time in which specific genotypes were observed, a detailed overview of which genotypes were most likely to be epidemiologically linked was determined. These combined data for epidemiologically linked clusters permitted the identification of 7 major clonal complexes extant during the epidemic. Significant geographical clustering of isolates by clonal complex was observed using at least one clustering metric for clonal complexes 1–5. Too few samples were available from clonal complexes 6 and 7 to make statistically robust inferences about geographic clustering. However, genetic and chronological data would suggest these isolates constitute two epidemiologically linked clusters. The occurrence and frequency of these clonal complexes and constituent 98 genotypes, varied over the study period (Fig 3). Sole use of minimum spanning trees largely recapitulated what combined epidemiological data and genotype information had suggested, with 82% of epidemiologically linked genotypes clustering together (see Fig 5). In particular, the largest cluster accounted for the majority of genotypes which were classified as belonging to clonal complexes 1, 2 and 6. Clonal complexes 3, 4 and 7 also exhibited clustering.

It is noteworthy that for a small minority of strain genotypes, clustering by genetic data alone resulted in clonal complex assignation that was not congruent with the epidemiological information. For example, strain 8 clustered using genetic data alone within clonal complex 2 whilst epidemiological data suggests it is part of clonal complex 1 (Fig 5 and S1 Dataset). This is likely the result of hyper variability in some loci affecting clustering by MST. In total, only 18% of genotypes failed to cluster consistently by MST when genotype data were used alone. In particular clonal complex 5 exhibited virtually no clustering based on single locus or double-locus variation. A common core of 6 loci with identical alleles was observed in most clonal complex 5 isolates with variation at the remaining 5 loci exhibiting the highest DIs. This hypervariation at multiple loci explains why MST clustering based on single locus / double-locus variation failed to cluster these isolates. However such cases were relatively rare and were easily assigned to their correct clonal complex upon combined use of location and chronology of infection epidemiological data.

Hierarchical clustering of MLVA genotype data alone by dendrogram revealed that the NI *B*. *abortus* population consisted of two distinct major lineages and constituent sub lineages (See Fig 4). None of the clonal complexes identified above were observed to cluster into discrete sublineages within the dendrogram. However, this is perhaps not surprising seeing as the dendrogram was constructed from purely genotypic data unlike the MST data which also took traditional epidemiological data into account. It is important to note however, that whilst clonal complex 5 isolates did not cluster by MST, they do in the dendrogram which makes use of the common core of 6 identical alleles to cluster them more effectively, indicating our designation of these isolates as closely related is correct. Sole reliance on genotype data from VNTR loci to infer epidemiological linkage is inadvisable owing to potential homoplasy / convergent evolution inherent in the loci [33–35]. Our findings are similar to those of Higgins *et al*., [21] who used MLVA to help elucidate the epidemiology of *Brucella abortus* in the United States. They recommended combining genotype data with epidemiological data describing geography and chronology of infection to maximise the value of the data.

This being said, the dendrogram clustering does still reveal some potentially interesting findings, at higher levels of hierarchical clustering relating to major lineages. Isolates from clonal complexes 1, 2, 3, 4 and 6 are all found in major lineage 1, whilst those from clonal complexes 5 and 7 are found in major lineage 2 (see Fig 4). MST data was also suggestive that clonal complexes 1, 2, 3, 4 and 6 were closely related owing to the fact that they clustered very closely (See Fig 5). Conversely MST data suggested that clonal complexes 5 and 7 were not closely related to the others. The dendrogram data are therefore quite congruent with MST data. Interestingly, clonal complex 7 was isolated in 1991 in a period just before putative eradication and the subsequent recrudescence, whilst clonal complex 5 was extant during the most recent epidemic. This perhaps indicates that the emergence of clonal complex 5 in recent times has some linkage to the past outbreak–perhaps evolving from a clonal complex 7 like ancestor? In addition, the fact that major lineage 1 and associated clonal complexes are so distinct from major lineage 2 implies that the recent epidemic was largely due to a secondary introduction of the pathogen and its subsequent diversification. These findings, if supported by additional genetic testing, are potentially extremely useful from an epidemiological perspective, and would not have been found using traditional, non molecular epidemiological approaches. It would however be beneficial to explore these hypotheses further with more robust phylogenetic inference from whole genome sequence data.

Interestingly, the biovar 2 samples (MLVA11 genotypes 37, 66 and 110) were all observed to cluster together in clonal complex 6. The fact they cluster together is encouraging as the shared biovar designation is indicative that they should be genetically similar—a fact confirmed by application of the MLVA11 panel described herein. These data suggest that epidemiological inferences based on clonal complex 6 data are robust. More interesting, however, is that these biovar 2 genotypes appear to be closely related to the biovar 1 samples from Clonal Complex 1. Indeed, clonal complex 6 is part of the larger meta cluster of biovar 1 strains making up clonal complexes 1,2, 3, 4 and 6 (see Figs 4 and 5). Such clustering perhaps appears counterintuitive as perhaps one would expect samples from biovar 2 to be genetically distinct, potentially lying within their own lineage. The clustering observed is due to allelic similarity at multiple MLVA11 loci, and whilst homoplasy / convergent evolution is a feature of such loci [33–35], the probability of 9 of 11 loci tested being identical by state rather than descent is low. Indeed homoplasy at an even greater number of markers would be an even rarer event. With the latter in mind, it is worth noting that our data indicates genetic homogeneity between biovar 1 and 2 isolates at 20 of the 26 loci from the full MLVA26 panel (See S3 Dataset). When the three most unstable loci from the MLVA26 panel are removed (Hoofprints 7, VNTR12a and VNTR12b) as can be justified because of their observed intra animal hypervariability (see previously), the remaining MLVA23 panel when subjected to eBurst analysis recapitulates the MST relationships between these isolates shown in Fig 5 (See S1 Fig). This is compelling empirical evidence that the biovar 1 and biovar 2 isolates are very genetically similar. We concede however that to address this issue properly within this epidemiological setting, more robust phylogenetic inference using genome sequencing and stable markers like single nucleotide polymorphisms (SNPs) will be necessary. It is of note however that other previously published findings have been similar to what we describe here. In particular, other MLVA studies have reported congruent results [4, 20]. More recently, Wattam *et al*., [36] have undertaken genome sequencing of multiple *Brucella abortus* isolates and observed that from 3 identifiable clades of *B*.*abortus*, isolates of biovar 1, 2 and 4 cluster within the same clade.

### MLVA11 within herds and within animals–locus hypervariability

Allelic variability underpins the usefulness of VNTR loci in molecular epidemiology. Ideally, the best markers mutate at a rate consistent with the epidemic. If loci mutate too slowly, incorrect inferences on epidemiological linkage can be made, whilst if mutation is too fast, true links are potentially missed or lost. Hyper-variability at some of the *Brucella* VNTRs has been observed previously. Kulakov *et al*., [37] have reported that in general, within clonal complex variability of genotype is a feature of lineages of *B*. *abortus*. Additionally, Whatmore *et al*., [4] reported that loci VNTR 12b, Hoofprint 5 and, as in this study, Hoofprints 7 were prone to hypervariability both within animal and post culture. Variability in some of the Hoofprints markers has also been noted before by Bricker *et al* [18]. Also, within the *Brucella* genus, a study reporting on an outbreak of *B*. *canis* at a Hungarian dog kennel, noted that high mutation rates of some MLVA loci within animals presented a difficulty in unambiguously inferring epidemiological relationships between animals [38]. Similar findings with hypervariable loci affecting precision have been noted in cattle [39].

Analyses conducted on sub-population B herd level data indicates that both the number of animals affected in a herd breakdown and the time taken to clear infection both result in increased diversity of strain genotypes. This is an intuitive finding since opportunity for mutation and production of new variants will be enhanced by a larger population of bacteria across multiple hosts which have time to diversify. Therefore, an efficient and effective eradication scheme which reduces both time to clear infection and opportunity to infect multiple animals should increase precision of molecular epidemiological methods. Whilst appealing and intuitive however, we do not have the data to confirm that any production of new variants is not occurring at the culture stage. Our data on within animal molecular types from sub-population D indicated that hyper-variability of some VNTR loci, in particular Hoofprint 3 and Hoofprint 4, gave rise to multiple VNTR types during the same outbreak. Analysis indicated a signficiant but weak correlation between number of infected sites sampled and diversity of strain population within animal. Again, this makes intuitive sense, however the number of new strains detected as a result of sampling so many extra sites was low indicating that whilst within host diversification does appear to occur, it was not occurring at a great rate. However we did not have adequate data to assess the effect of time on diversification within animals as this would have required data on exact time of infection, a metric difficult to determine from field data. Conceivably, if animals were identified quickly after infection, as seems likely for data set D which was collected late in the epidemic, perhaps there was insufficient time for the pathogen to produce more diversity. By way of contrast, if within animal diversification is genuinely not a major issue, then as analysis of sub-population B suggests, is diversification happening during transmission to new hosts? In truth, with the data available, we are limited in what we can say about when mutation has occurred, principally because we cannot rule out that potentially, new variants have arisen in the culture process. The latter phenomenon has been documented for the MLVA16 panel [20], investigation of a *B*. *abortus* outbreak in Brazilian cattle found that MLVA profiles were stable *in vivo* but exhibited considerable change *in vitro* [40]. In future, in light of the analyses described, it would be beneficial to attempt to determine the stability of local MLVA genotypes in culture. Regardless of when or where mutations occurred, hyper-variability of loci resulting in a lack of stability of genotype within individual breakdowns could conceivably affect epidemiological precision. Encouragingly however, as with sub-population D in this study, provided systematic sampling of multiple animals and multiple infected sites within individual animals is undertaken, it becomes obvious that divergent genotypes still share a common core of less variable loci with alleles identical by descent. Consequently, when combined with epidemiological data, divergent genotypes could be associated with the correct clonal complex and then linked to related outbreaks on the basis of shared clonal complex assignation. This underlines the importance of undertaking systematic surveys of population diversity in outbreaks of *B*. *abortus*. Such an approach serves to maintain the transparency of epidemiological links between outbreaks.

### Herd-level epidemiology

From the period 1991–2002, clonal complexes 1, 2, 4, 5, 6 and 7 were present in the population and account for 79 disclosing isolates. Clonal complex 7, comprising 5 MLVA11 profiles, was identified only in 1991, and is perhaps a relic of a previous outbreak that was subsequently not observed in the recrudescence of infection from 1997 onwards. Data from these early years are limited however due to a shortage of samples stored. More encouragingly however, for all isolates obtained, host animal identities are known, and movement histories can be tracked using APHIS [29]. These complementary cattle movement data potentially make the MLVA data a more powerful tool for investigating the sources of infection and their spread. However, we identify some local constraints on the complementary use of such data below.

Interestingly, the earlier evolved clonal complexes (clonal complexes 1, 2, 4, 5 and 6) exhibited the most widespread distribution across Northern Ireland during the height of the epidemic between 2003 and 2008 (See Figs 2 and 3). The latter is probably due to the fact that these early evolved complexes had the opportunity to spread before the reactive efforts of the eradication scheme gathered momentum.

At the height of the recent epidemic (2003–2008) clonal complexes 1, 2, 3, 4, 5 and 6 were all observed accounting for 257 individual herd breakdowns. However clonal complexes 4, 5 and 6, disappeared from the population, seemingly becoming extinct after 2004, having only accounted for a small number of total disclosing isolates / breakdowns (20 isolates – 7.7%). At this peak of the epidemic, the majority of cases (n = 132–51%) were caused by clonal complex 1. After this peak from 2009–2012, clonal complexes 2 and 3 became extinct, leaving only clonal complex 1 extant. These findings are consistent with the effectiveness of the brucellosis eradication scheme succeeding in purging most clonal complexes throughout 2004–2008, such that by 2009 only clonal complex 1 was extant, but with much reduced diversity compared to previous years, a fact illustrated by the MLVA11 diversity indices in the bottom row of Table 2 showing the diversity of the *B*. *abortus* population has been severely reduced from 0.947 to 0.634 in this period.

The later evolved clonal complex 3 presents an interesting case study in terms of more detailed epidemiological analysis, not least because in addition to the molecular characterisation of this “mini epidemic”, an independent classical veterinary epidemiological investigation was also conducted contemporaneously in the region it dominated [6]. This clonal complex was only extant during the period from 2004–2007 and remained tightly clustered in the Armagh DVO area, with 30 (86%) of the 35 isolates observed found in that DVO in this period (see Fig 3 and Table 3). Some apparent spread occurred in 2006 to other areas, however the vast majority of the isolates were retained in the Armagh DVO area. Perhaps as a result of its comparatively late appearance, and the well established eradication scheme being in place, this clonal complex was well contained and controlled.

The molecular data presented here are largely congruent with the results of the classical veterinary epidemiological investigation carried out by Abernethy *et al* [6] referred to above. In the latter study, a subset of 41 herd breakdowns from December 2005 to September 2006 in the Armagh DVO area were studied in detail. Abernethy *et al* [6] concluded that approximately 71% of these breakdowns were epidemiologically linked, with the most likely explanation being contiguous spread between neighbouring farms by cattle to cattle contact at field interfaces. The remaining 29% of herd breakdowns observed were not linked to the latter tightly clustered herds.

Molecular data for this time period and location indicate that only two clonal complexes were active in the Armagh DVO area–clonal complexes 2 and 3 (See Table 3). In total, 27 disclosing Isolates from this region were available from this time period. The disparity of 41 herd breakdowns and only 27 disclosing isolates is probably accounted for by the fact that whilst serological testing can detect herd breakdowns, not all breakdowns will produce *B*. *abortus* cultures that can be genotyped. Of the 27 disclosing isolates, 21 (78%) were found to belong to clonal complex 3 and exhibited tight clustering in the same area of Armagh DVO that Abernethy *et al* [6] identified (See Fig 2). These data are consistent with the tightly clustered herds in question being epidemiologically linked as had been inferred by Abernethy *et al* [6]. The remaining 6 isolates (22%) were from clonal complex 2 and therefore not epidemiologically linked to the tightly clustered herds accounting for the majority of the herd breakdowns observed in Armagh DVO over this time period.

It is worth noting that 5 isolates of Clonal Complex 3 were noted outside of the Armagh DVO area and the cluster associated with contiguous spread–see Fig 2B. With two of these isolates, associated animal movement data can help to inform on likely sources and spread of disease. For isolate number 2 as shown on Fig 2B, the associated herd succumbed to disease in 2006, and had previously been subject to animal movement from a previously brucellosis affected herd with an identical strain genotype in 2004. Similarly, for isolate number 5 as shown on Fig 2B, the associated herd succumbed to disease in 2005 and was subsequently found to have purchased animals from a herd within the Armagh DVO cluster, in which brucellosis was detected in 2006. In the latter source herd, a closely related single locus variant of isolate number 5 was found.

The remaining 3 isolates outside Armagh DVO could not be traced back, using animal movement records, to putative sources also exhibiting Clonal Complex 3. This failure to detect an infection source may be linked to two contributing phenomena. The first, relates to a feature of farm management and animal husbandry in Northern Ireland. Local farm enterprises are considerably atomised with land parcels distant from home farms used to graze animals. Animals belonging to such herds which utilise this land are however recorded as inhabiting a single point location linked to the home farm buildings. As a result, it is feasible that small scale, unrecorded, animal movements to outlying land parcels could have brought animals into contiguous contact with infected animals from different herds, thereby facilitating spread of disease. Alternatively, during this outbreak, it was noted that there was potential criminal involvement in the deliberate spread of disease via dumping of aborted foetuses on farmland previously unaffected by brucellosis. Whilst rare, this is another potential disease transmission mechanism whose source validation could not have been tracked by recorded animal movement data.

## Conclusions

Our findings demonstrate the usefulness of the MLVA technique and the importance of structured population sampling. MLVA, in conjunction with basic epidemiological data can inform on the relatedness of isolates of *B*. *abortus* and is of benefit in elucidating sources of infection, linked breakdowns, monitoring of disease spread and more generally in the management of brucellosis eradication schemes. An example of this is our ability to trace likely sources of Clonal Complex 3 related infection back to source herds in the Armagh DVO cluster.

The hyper-variable nature of some loci is an area of concern since multiple genotypes related to single outbreaks can negatively impact an epidemiological investigation. Consequently, very fine epidemiological precision determining links between herds on the basis of shared individual MLVA genotypes was difficult to assess. However, we have demonstrated that provided a systematically structured sampling of multiple animals within outbreak affected herds and individual animals is undertaken to catalogue the allelic variation occurring, epidemiological precision can be maintained. Novel variants can still be assigned to specific clonal complexes on the basis of a shared core of specific alleles at specific loci. These clonal complex data can be used to successfully determine which herds are epidemiologically linked, thereby informing on the likely spread of disease from originating sources. Encouragingly, our molecular data confirm the findings of an independent conventional epidemiological study [6] providing further evidence for the usefulness of MLVA.

In future, a complementary approach could be the use of bacterial whole-genome sequencing to identify very stable and unidirectional micro evolutionary events such as single nucleotide mutations which could also inform on transmission chains and dynamics at a much finer level. These phylo-dynamic approaches have been successful when applied to human pathogens such as in the recent outbreak of *E*. *coli* O104:H4 in Germany [41], *M*. *tuberculosis* in the United Kingdom [42] and interestingly, from an animal health perspective, in bovine and badger hosts of *Mycobacterium bovis* in Northern Ireland [43]. Such approaches may also help to further elucidate the evolutionary history of the pathogen in Northern Ireland thereby informing on potential links to previous epidemics and possible sources.

## Supporting Information

S1 Dataset Genotype and DVO location information for all 1270 *B*. *abortus* strain isolates. (XLSX)

S2 Dataset Spatial clustering statistics outputs from ARC GIS Ripley’s K Function applied to 7 clonal complex first isolates. (XLSX)

S3 Dataset MLVA26 data from closely clustered Clonal Complex 1 and Clonal Complex 6 (biovars 1 and 2 respectively) isolates. (XLSX)

S1 Fig MLVA23 Minimum Spanning Tree drawn by eBurst for closely clustered Clonal Complex 1 and Clonal Complex 6 (biovars 1 and 2 respectively) isolates. (TIF)

S1 Table Spatial clustering statistics outputs from ARC GIS nearest neighbour analysis applied to 7 clonal complex first isolates. (DOCX)

## References

[1] Foster JT, Beckstrom-Sternberg SM, Pearson T, Beckstrom-Sternberg JS, Chain PSG, Roberto FF, et al (2009) Whole genome based phylogeny and divergence of the genus *Brucella* . J Bacteriol 191: 2864–2870. 10.1128/JB.01581-08 19201792 PMC2668414

[2] Adone R, Pasquali P (2013) Epidemiosurveillance of brucellosis. Rev Sci Tech. 32: 199–205. 23837377 10.20506/rst.32.1.2202

[3] Crawford RP, Huber JD, Adams BS (1990) Epidemiology and Surveillance In: Nielsen K, Duncan JR, Editors, Animal Brucellosis. Boca Raton, CRC Press pp. 131–151.

[4] Whatmore AM, Shankster SJ, Perrett LL, Murphy TJ, Brew SD, Thirlwall RE, et al (2006) Identification and Characterization of Variable Number Tandem repeat Markers for Typing of *Brucella* spp. J Clin Microbiol 44: 1982–1993. 16757588 10.1128/JCM.02039-05 PMC1489437

[5] Whatmore AM (2009) Current understanding of the genetic diversity of *Brucella* an expanding genus of zoonotic pathogens. Infect Genet Evol 9: 1168–1184. 10.1016/j.meegid.2009.07.001 19628055

[6] Abernethy DA, Moscard-Costello J, Dickson E, Harwood R, Burns K, McKillop E et al (2011) Epidemiology and management of a bovine brucellosis cluster in Northern Ireland. Prev Vet Med 98: 223–229. 10.1016/j.prevetmed.2010.11.002 21144605

[7] Diaz Aparicio E (2013) Epidemiology of brucellosis in domestic animals caused by *Brucella melitensis*, *Brucella suis* and *Brucella abortus* . Rev Sci Tech 32: 53–60. 23837364

[8] Poester FP, Samartino LE, Santos RL (2013) Pathogenesis and pathobiology of brucellosis in livestock. Rev Sci Tech 32: 105–115. 23837369 10.20506/rst.32.1.2193

[9] Godfroid J, Garin-Bastuji B, Saegerman C, Blasco JM (2013) Brucellosis in terrestrial wildlife. Rev Sci Tech 32: 27–42. 23837363 10.20506/rst.32.1.2180

[10] Ragan V, Vroegindewey G, Babcock S (2013) International standards for brucellosis prevention and management. Rev Sci Tech 32: 189–198. 23837376 10.20506/rst.32.1.2203

[11] OIE Manual of diagnostic tests and vaccines for terrestrial animals Chapter 2.4.3 Bovine Brucellosis. Available: http://www.oie.int/fileadmin/Home/eng/Health_standards/tahm/2.04.03_BOVINE_BRUCELL.pdf. Accessed 22 September 2014.

[12] EU council directive 64/432/EEC on animal health problems affecting intra-Community trade in bovine animals and swine. Available: http://eur-lex.europa.eu/LexUriServ/LexUriServ.do?uri=CELEX:31964L0432:EN:NOT. Accessed 22 September 2014.

[13] Scott JC, Koylass MS, Stubberfield MR, Whatmore AM (2007) Multiplex assay based on single nucleotide polymorphisms for rapid identification of *Brucella* isolates at the species level. Appl Environ Microbiol 73: 7331–7337. 17890328 10.1128/AEM.00976-07 PMC2168218

[14] OIE world animal health information database. Available: http://www.oie.int/wahis/public.php. Accessed 19 September 2014.

[15] Stringer LA, Guitian FJ, Abernethy DA, Honhold NH, Menzies FD (2008). Risk associated with animals moved from herds infected with brucellosis in Northern Ireland. Prev Vet Med 84: 72–84. 10.1016/j.prevetmed.2007.11.005 18207262

[16] Abernethy DA, Pfeiffer DU, Watt R, Denny GO, McCullough S, McDowell SW (2006) Epidemiology of bovine brucellosis in Northern Ireland between 1990 and 2000. Vet Rec 158: 717–721. 16731701 10.1136/vr.158.21.717

[17] Leiser OP, Corn JL, Schmit BS, Keim PS, Foster JT (2013) Feral swine brucellosis in the United States and prospective genomic techniques for disease epidemiology. Vet Microbiol 166: 1–10. 10.1016/j.vetmic.2013.02.025 23548760

[18] Bricker BJ, Ewalt DR, Halling SM (2003) *Brucella* ‘HOOF-Prints’: strain typing by multi locus analysis of variable number tandem repeats (VNTRs). BMC Microbiol 3: 15 12857351 10.1186/1471-2180-3-15 PMC183870

[19] Skuce RA, Mallon TR, McCormick CM, McBride SH, Clarke G, Thompson A, et al (2010) *Mycobacterium bovis* genotypes in Northern Ireland: herd level surveillance (2003 to 2008). Vet Rec 167: 684–689. 10.1136/vr.c5108 21257483

[20] Le Flèche P, Jacques I, Grayon M, Al Dahouk S, Bouchon P, Denoeud F, et al (2006) Evaluation and selection of tandem repeat loci for a *Brucella* MLVA typing assay. BMC Microbiol 6: 9 16469109 10.1186/1471-2180-6-9 PMC1513380

[21] Higgins J, Stuber T, Quance C, Edwards WH, Tiller RV, Linfield T, et al (2012). Molecular epidemiology of *Brucella abortus* isolates from cattle, elk and bison in the United States, 1998 to 2011. Appl Environ Microbiol 78: 3674–3684. 10.1128/AEM.00045-12 22427502 PMC3346378

[22] Garofolo G, Di Gianntale E, De Massis F, Zilli K, Ancora M, Camma C, et al (2013). Investigating the genetic diversity of *Brucella abortus* and *Brucella melitensis* in Italy with MLVA-16. Infect Genet Evol 19: 59–70. 10.1016/j.meegid.2013.06.021 23831636

[23] Kiliç S, Ivanov IN, Durmaz R, Bayraktar MR, Ayaslioglu E, Uyanik MH, et al (2011) Multiple locus variable number tandem repeat analysis genotyping of human *Brucella* isolates from Turkey. J Clin Microbiol 49: 3276–3283. 10.1128/JCM.02538-10 21795514 PMC3165627

[24] Gwida M, Neubauer H, Ilhan Z, Schmoock G, Melzer F, Nockler K, et al (2012) Cross-border molecular tracing of Brucellosis in Europe. Comp Immunol Microbiol Infect Dis 35: 181–185. 10.1016/j.cimid.2011.12.012 22277829

[25] Jiang H, Fan M, Chen J, Mi J, Yu R, Zhao H, et al (2011). MLVA genotyping of Chinese human *Brucella melitensis* biovar 1, 2 and 3 isolates. BMC Microbiol 11: 256 10.1186/1471-2180-11-256 22108057 PMC3233519

[26] DARD–Department of Agriculture and Rural Development Northern Ireland–monthly brucellosis statistics. Available: http://www.dardni.gov.uk/index/statistics/animal-disease-statistics/statistics-brucellosis.htm. Accessed 22 September 2014.

[27] Alton GG, Jones LM, Angus RD, Verger JM, editors (1988) Techniques for the Brucellosis laboratory Paris Institute National de la Recherche Agronomique pp.13–61.

[28] Scholz HC, Vergnaud G (2013) Molecular characterisation of *Brucella* species. Rev Sci Tech 32: 149–162. 23837373 10.20506/rst.32.1.2189

[29] Houston R (2001) A computerised database system for bovine traceability. Rev Sci Tech 20: 652–661. 11548534 10.20506/rst.20.2.1293

[30] Hunter PR, Gaston MA (1988) Numerical index of the discriminatory ability of typing systems: an application of Simpson’s index of diversity. J Clin Microbiol 26: 2465–2466. 3069867 10.1128/jcm.26.11.2465-2466.1988 PMC266921

[31] Spratt BG, Hanage WP, Li B, Aanensen DM, Feil EJ (2004) Displaying the relatedness among isolates of bacterial species—the eBURST approach. FEMS Microbiol Lett 241: 129–34. 15598523 10.1016/j.femsle.2004.11.015

[32] Dixon PM (2002) Ripley’s K Function, In: El-Shaarawi AH and Piegorsch WW, Editors, Encyclopedia of Environmetrics. John Wiley and Sons pp. 1796–1803.

[33] Comas I, Homolka S, Niemann S, Gagneux S (2009) Genotyping of genetically monomorphic bacteria: DNA sequencing in *Mycobacterium tuberculosis* highlights the limitations of current methodologies. PLOS One 4: e7815 10.1371/journal.pone.0007815 19915672 PMC2772813

[34] Pearson T, Okinaka RT, Foster JT, Keim P (2013) Phylogenetic understanding of clonal populations in an era of whole genome sequencing. Infect Genet Evol 9: 1010–1019. 10.1016/j.meegid.2009.05.014 19477301

[35] Reyes JF, Chan CHS, Tanaka MM (2012) Impact of homoplasy on variable number of tandem repeats and spoligotypes in *Mycobacterium tuberculosis* . Infect Genet Evol 12: 811–818. 10.1016/j.meegid.2011.05.018 21683165

[36] Wattam AR, Foster JT, Shrinivasrao PM, Beckstrom-Sternberg SM, Beckstrom-Sternberg JM, Dickerman AW, et al (2014) Comparative Phylogenomics and Evolution of the *Brucellae* Reveal a Path to Virulence. J. Bacteriol 196: 920–930. 10.1128/JB.01091-13 24336939 PMC3957692

[37] Kulakov Y, Zheludkov MM, Sclyarov OD (2010) Variable number tandem repeat markers for identification of *Brucella abortus* 82 and 75/79-AV vaccine strains. Vaccine 28S: F41–F45. 10.1016/j.vaccine.2010.03.051 20362200

[38] Gyuranecz M, Rannals BD, Allen CA, Janosi S, Keim P, Foster JT (2013) Within host evolution of *Brucella canis* during a canine brucellosis outbreak in a kennel. BMC Vet Res 9: 76 10.1186/1746-6148-9-76 23587163 PMC3637509

[39] Alvarez J, Saez JL, Garcia N, Serrat C, Perez-Sancho M, Gonzalez S, et al (2011) Management of an outbreak of brucellosis due to *B*. *melitensis* in dairy cattle in Spain. Res Vet Sci 90: 208–211. 10.1016/j.rvsc.2010.05.028 20579679

[40] Dorneles EM, Santana JA, Alves TM, Pauletti RB, Mol JP, Heinemann MB, et al (2014) Genetic stability of *Brucella abortus* isolates from an outbreak by multiple locus variable tandem repeat analysis (MLVA16). BMC Microbiol 14: 186 10.1186/1471-2180-14-186 25015840 PMC4112982

[41] Mellman A, Harmsen D, Cummings CA, Zertz EB, Leopold SR, Rico A, et al (2011) Prospective genomic characterisation of the German enterohemorrhagic *Escherichia coli* O104:H4 outbreak by rapid next generation sequencing technology. PLOS One 6(7) e22751 10.1371/journal.pone.0022751 21799941 PMC3140518

[42] Walker TM, Ip CL, Harrell RH, Evans JT, Kapatai G, Deadicoat MJ, et al (2013) Whole-genome sequencing to delineate *Mycobacterium tuberculosis* outbreaks: a retrospective observational study. Lancet Infect Dis 13:137–46 10.1016/S1473-3099(12)70277-3 23158499 PMC3556524

[43] Biek R, O’Hare A, Wright D, Mallon T, McCormick C, Orton RJ, et al (2012) Whole genome sequencing reveals local transmission patterns of *Mycobacterium bovis* in sympatric cattle and badger populations. PLOS Pathog 8(11): e1003008 10.1371/journal.ppat.1003008 23209404 PMC3510252

