# Supplementary figures and images for: Molecular Epidemiology of Brucella abortus in Northern Ireland—1991 to 2012
Source: PLoS One. 2015 Sep 1;10(9):e0136721. doi: 10.1371/journal.pone.0136721 (PMC4556700; doi:10.1371/journal.pone.0136721)

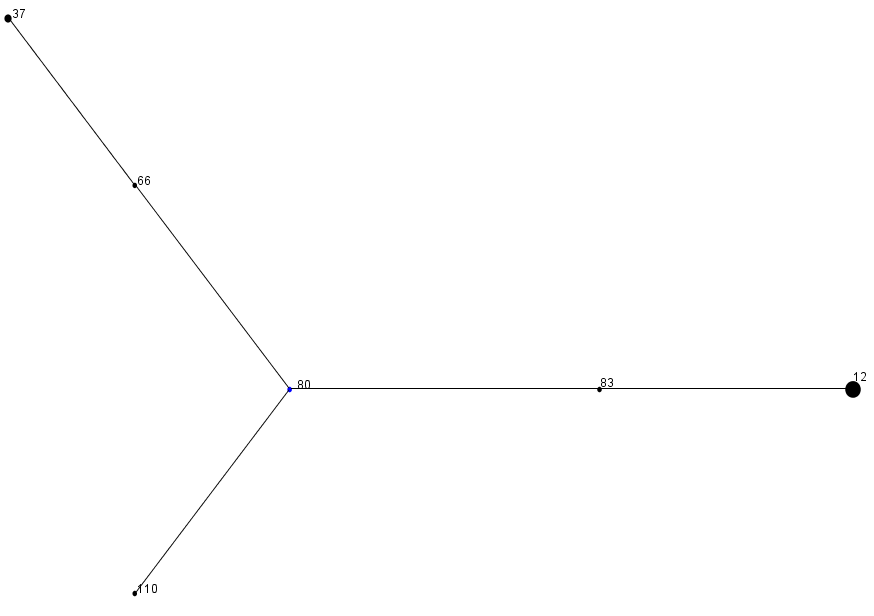

Supplement: S1 Fig — (TIF) [file pone.0136721.s004.tif]
