# Supplementary material for: Molecular Epidemiology of Brucella abortus in Northern Ireland—1991 to 2012
Source: PLoS One. 2015 Sep 1;10(9):e0136721. doi: 10.1371/journal.pone.0136721 (PMC4556700; doi:10.1371/journal.pone.0136721)
Supplement: S1 Table — (DOCX) [file pone.0136721.s005.docx]

| **Clonal Complex** | **Expected Mean Distance** | **Observed Mean distance** | **Z score** | **P value** |
| --- | --- | --- | --- | --- |
| **1** | 3971.9 | 2149.5 | -13.019454 | 0.000000 |
| **2** | 6107.6 | 3136.9 | -8.004554 | 0.000000 |
| **3** | 2673.1 | 2607.7 | -0.277026 | 0.781760 |
| **4** | 8102.7 | 3737.9 | -5.048513 | 0.000000 |
| **5** | 3496.6 | 2252.9 | -3.263353 | 0.001101 |
| **6** | 6182.0 | 16085.0 | 6.852461 | 0.000000 |
| **7** | 10717.9 | 19864.3 | 3.650469 | 0.000262 |

Nearest neighbour analysis outputs by Clonal Complex.
